# The Institutional Worker

**Published:** 1911-12-23

**Authors:** 


THE
### INSTITUTIONAL WORKER
**Being a Special Supplement to "The Hospital"**

### Editor's Notices.

**Contributions:**

Contributions should be written, or preferably typed, on one side of the paper only, and all articles sent in are accepted upon the distinct understanding that they are forwarded to The Hospital only.

The Editor cannot undertake to return MSS. not used, but when a stamped directed envelope is enclosed the MSS. may be returned if a special request is made.

Accepted articles and paragraphs of news will be paid for after publication at the scale rate.

**Address:**

To prevent delay all contributions and letters on editorial business must be addressed exclusively to the Editor, "The Hospital," 29 Southampton Street, Strand, London, W.C. It is important that this regulation shall be strictly observed.

**Correspondence:**

Correspondence on all subjects is invited. The name and address of correspondents must be given as a guarantee of good faith, but not necessarily for publication.

**Special Articles:**

Special articles are invited, and questions, inquiries, and paragraphs upon all matters relating to the work, administration, and management of general, special, mental, fever, cottage and convalescent hospitals, sanatoria, homes, institutions, societies and organisations for the treatment or care of the sick, injured and dependents of all classes. Every matter affecting the interests and welfare of the staff of all grades working in these institutions will receive special consideration.

**Administrative Medicine, Research and Items of News:**

Special terms will be paid for approved articles on subjects in this wide field by experts who have made some section of it their special study and interest. We wish to give prominence to every movement tending to advance accuracy of diagnosis, efficiency in treatment, and the development of modern methods to eradicate disease and promote the welfare of the suffering.

**A Bureau of Information:**

We invite inquiries and applications for information and help in providing for that numerous class of sufferers who require special care or house-room which they cannot obtain in their own homes or secure for themselves. We cannot, however, prescribe or recommend practitioners.

**Books for Review:**

Publishers are particularly requested to send advance proofs of any new books of importance whenever possible, as the Editor has made arrangements to publish immediate reviews on a new plan.

**Photographs, Plans, Blocks, and Illustrations:**

It is requested that wherever possible MSS. may be accompanied by illustrations in any of the above forms. The name of the sender and of the article to which it belongs should be written on the back of each photograph, drawing, or block for purposes of identification.

**Local Papers:**

Newspapers containing reports or news paragraphs should be marked and addressed to the Sub-Editor.

**The Coupon System:**

A coupon will be found at the bottom of the third inside page of the cover each week. This coupon must be attached to every question or inquiry to which an answer is desired in The Hospital.

### Manager's Notices.

Letters relating to the publication, sale, and advertising departments of The Hospital must be addressed to the Manager, "The Hospital," The Hospital Building, 28 and 29 Southampton Street, Strand, London, W.C.

**Subscriptions** which may begin at any time, are payable in advance. Cheques and Post-Office orders, crossed London County and Westminster Bank, Covent Garden branch, should be made payable to the Manager of The Hospital and addressed to him at The Hospital Building, 28 and 29 Southampton Street, Strand, London, W.C.

**Advertisements:**

All advertisements for The Hospital must reach the Manager not later than Wednesday morning in each week. For scale of charges see pages v and 2.

**Classified Advertisements:**

All small advertisements will be classified, for this section of the paper is widely read and of special interest. We wish to encourage those wanting appointments who are attracted to institutional work, and therefore offer them the reduced rate of *twenty-one words and under for* 1s., and *for every ten and part of ten words beyond*, 6*d*.

### POINTS FOR INSTITUTION AUTHORITIES.

The economical management of an Institution depends very greatly upon purchasing supplies in the cheapest market.

It is often impossible locally to secure the highest quality at the lowest prices.

In consequence, a section will be reserved in this part of the paper for announcements of Institutions inviting Tenders foı Contracts.

This will enable Authorities of Institutions to secure tenders for supplies from all over the country, and ensure purchases at the lowest possible prices consistent with quality.

The charge for Tenders is 6s. per inch, and for this small outlay many pounds may be saved on supplies of all descriptions.

**Special Rates** will be quoted for those institutions which agree to send all their vacancy, contract, and public notices to The Hospital each year, so as to make it a file of such announcements for ready reference.

### POINTS FOR TRADE ADVERTISERS.

The Hospital is the organ of Institutions, whether of a Voluntary Character or those under Local Government, Poor Law and Municipal Authorities.

It is read by all Executive Officials and those responsible for the Construction, Management, and Equipment of Public Establishments.

It is also the Journal of the worker engaged in the Medical, Health and Preventive services.

There is no medium more appropriate and effective in the promotion of business in these directions than The Hospital.

### POINTS FOR INSTITUTIONAL WORKERS.

A very large number of Appointments Vacant appear every week in the columns of The Hospital.

If you cannot find a position suited to your requirements, make your wishes known through our Situations Wanted Column.

### Appointments Vacant.

### APPOINTMENTS VACANT.

### Poor-Law Vacancies.

### Appointments Vacant.

ASSISTANT MASTER (£40), Northampton Union. Mr. W. Fawkes, C. to G., Northampton. Dec. 28.

ASSISTANT MASTER (£32), Hunslet Union. Mr. F. W. Mee, C. to G., Hunslet. Dec. 27.

ASSISTANT MASTER (£30), Burton-on-Trent Union. Mr. C. F. Chamberlin, C. to G., Burton-on-Trent. Dec. 30.

ASSISTANT MEDICAL OFFICER (£80), Manchester Royal Infirmary.

ASSISTANT NURSE (£22), Congleton Union. Mr. H. Ferrand, C. to G., Sandbach. Dec. 29.

ASSISTANT NURSE (£30), Cuckfield Union. Mr. E. J. Waugh, C. to G., Hayward's Heath. Jan. 4.

ASSISTANT NURSE (£25), Wortley Union. Mr. J. Morton, C. to G., Grenoside, Sheffield.

ASSISTANT NURSE (£25), King's Lynn Union. Mr. R. C. Coulton, C. to G., King's Lynn.

ASSISTANT NURSE (£25), Bedford Union. Mr. W. Payne, C. to G., Bedford. Dec. 23.

ASSISTANT NURSES (£22), Sevenoaks Union. Mr. F. H. Vibart, C. to G., Bank Chambers, Sevenoaks. Jan. 1.

ASSISTANT WARRANT OFFICER (£80), Pontypridd Union. Mr. W. Spickett, C. to G., Pontypridd. Dec. 29.

BANDMASTER (£100, non-resident), St. Pancras Guardians. Mr. J. E. P. Hall, C. to G., Town Hall, Pancras Road, N.W. Dec. 27.

CHARGE NURSE (£30), Richmond Union. Mr. P. Umney, C. to G., Parkshot, Richmond, Surrey. Jan. 10.

CHARGE NURSE (£30), Rochford Union. F. Gregson, C. to G., 46 Alexandra Street, Southend-on-Sea. Dec. 28.

CHIEF MALE ATTENDANT (£35), Southampton Union. Mr. A. J. Walden, C. to G., Southampton. Dec. 23.

DAY CHARGE NURSE (£30), Guildford Union. W. S. V. Cullerne, C. to G., Commercial Road, Guildford.

FEMALE ATTENDANT (£20), Tendring Union. Matron of the Workhouse, Weeley, Essex.

FOSTER-MOTHER (£25), Ecclesall Bierlow Union. Mr. J. E. Moulding, C. to G., The Edge, Sheffield. Dec. 27.

FOSTER-MOTHER (£25), York Union. Mr. G. Sykes, C. to G., York. Dec. 29.

FOSTER-MOTHER (£30), Burnley Union. Mr. J. S. Horn, C. to G., Burnley. Jan. 3.

FOSTER-MOTHER (£28), Greenwich Union. Mr. S. Saw, C. to G., East Greenwich. Jan. 4.

HEAD MALE LUNATIC ATTENDANT (£40), MALE LUNATIC ATTENDANT (£30), Birkenhead Union. Mr. J. Carter, C. to G., Birkenhead. Dec. 25.

INFANTS' CHIEF CARETAKER (£28), York Union. Mr. G. Sykes, C. to G., York. Dec. 26.

INFANTS' NURSE (£25), Bucklow Union. Mr. G. Leigh, C. to G., Knutsford. Dec. 27.

JUNIOR ASSISTANT NURSES (two) (£22), Totnes Union. Mr. F. K. Windeatt, C. to G., 19 High Street, Totnes. Dec. 28.

MALE ATTENDANT (£26), Islington Guardians. Master of the Workhouse, St. John's Road, Upper Holloway, N.

MALE IMBECILE ATTENDANT (£26), Lincoln Union. Mr. W. B. Danby, C. to G., Lincoln.

MALE WARD ATTENDANT (£25), Cheltenham Union. Mr. J. Meek, C. to G., Cheltenham.

MASTER'S ASSISTANT CLERK (£25), Hackney Union. Master of the Workhouse, Sidney Road, Homerton, N.E.

MASTER AND MATRON (£100), Grimsby Union. Mr. J. F. Wintringham, C. to G., Great Grimsby. Jan. 1.

MASTER'S CLERK (£35), Newport (Mon.) Union. Mr. A. H. Rees, C. to G., Newport (Mon.). Dec. 27.

MIDWIFE (£35), Marylebone Guardians. Matron of the Workhouse, Northumberland Street, Marylebone Road, W.

Night Nurse (£65, non-resident), Holywell Union. Mr. P. H. Roberts, C. to G., Holywell. Dec. 29.

Night Sister (£40), Brighton Guardians. Mr. B. Burfield, C. to G., Parochial Offices, Brighton. Jan. 2.

Night Sister and Assistant Superintendent Nurse (£36), Newport (Mon.) Union. A. H. Rees, C. to G., Union Offices, Queen's Hill, Newport (Mon.). Jan. 10.

Night Superintendent (£40), Ecclesall Bierlow Union. J. E. Moulding, C. to G. Union Offices, The Edge, Sheffield. Jan. 2.

Night Superintendent and Masseuse (£38), Brentford Union. Mr. W. Stephens, C. to G., Union Offices, Isleworth, W. Jan. 2.

Nurse (£20), St. Giles and Bloomsbury Guardians. Mr. J. Appleton, C. to G., Broad Street, Bloomsbury, W.C. Dec. 30.

Nurse (£35), Tendring Union. Matron of the Workhouse, Weeley, Essex.

Nurses (£30), Gateshead Union. G. Craighill, C. to G. Poor Law Union Offices, Gateshead. January 6.

Nursery Attendant (£25), Marylebone Guardians. Matron of the Workhouse, Northumberland Street, Marylebone Road, W.

Probationer (£12), Kettering Union. Mr. E. R. Lane, Acting C. to G., Poor Law Offices, George Street, Kettering. Dec. 28.

Probationer (£10), North Bierley Union. Mr. W. G. Cooper, C. to G., 4 Town Hall Street, Bradford. Dec. 30.

Probationer (£10), Bromyard Union. Mr. F. W. Nicholl, C. to G., Bromyard. Jan. 1.

Probationer (£13), West Ham Union. Mr. T. Smith, C. to G., Leytonstone, N.E.

Probationer Nurses (£10), Burnley Union. Mr. J. S. Horn, C. to G., Union Offices, Burnley.

Probationer Nurse (£5), Mansfield Union. W. S. Cockerham, C. to G., 105 Stockwell Gate, Mansfield. Dec. 30.

Sisters (£30), Kensington Guardians. W. R. Stephens, C. to G., Marloes Road, Kensington.

Sister in Charge of Mental Wards (£40), Nottingham Guardians. Mr. G. M. Howard, C. to G., Nottingham. Dec. 23.

Staff Nurse (£24), Holborn Union. Matron of the Workhouse, 1a Shepherdess Walk, N.

Staff Nurses (£24), Wandsworth Union. F. W. Piper, C. to G., St. John's Hill, Wandsworth, S.W.

Tailoring Attendant (£35), St. Pancras Guardians. Mr. J. E. P. Hall, C. to G., Town Hall, Pancras Road, N.W.

Valuer for Special Properties, Malling Union. Mr. F. J. Allison, C. to G., West Malling. Dec. 27.

Ward Sister (£30), Bermondsey Guardians. Matron of the Infirmary, Lower Road, Rotherhithe.

### Situations Vacant.

Assistant Laundress (18s. weekly, non-resident), Islington Guardians. Matron of the Infirmary, Highgate Hill, Upper Holloway, N.

Cook, &c. (£30), Huddersfield Union. Mr. E. A. Rigby, C. to G., Huddersfield.

Cook-General (£23), Eastbourne Union. The Matron of the Workhouse, Eastbourne.

Female Cook (£25), Chesterton Union. Mr. J. F. Symonds, C. to G., Cambridge. Dec. 29.

Laundress (£25), Colchester Guardians. Mr. C. E. White, C. to G., Colchester. Dec. 28.

Laundress (£25), Holbeach Union. Mr. S. S. Mossop, jun., C. to G., Holbeach.

Male Cook (£40), Leeds Union. Mr. J. H. Ford, C. to G., Leeds. Dec. 30.

Porter (£25), Colchester Guardians. Mr. C. E. White, C. to G., Colchester. Dec. 28.

Stoker and Washing Machine Attendant (£32 12s.), Islington Guardians. Headmaster of the Schools, Hornsey Road, N.

Working Laundress (£20), Hertford Union. Matron of the Workhouse, Hertford. Dec. 29.

### Institutional and Administrative Vacancies.

### Appointments Vacant.

Matron (£60), Putney Hospital. Secretary, 198 Upper Richmond Road, Putney, S.W. Dec. 31.

Matron (£63), Royal Albert Hospital, Devonport. Captain C. W. Dickinson, R.N., Secretary. Jan. 10.

### Municipal Vacancies.

### Appointments Vacant.

Assistant Inspector of Nuisances and Health Visitor (£100), Borough of Stafford. Mr. R. Battle, Town Clerk, Borough Hall, Stafford. Jan. 8.

Assistant Nurse (£30), Hampton U.D. Council. E. Cozens, Clerk, Public Offices, Hampton, Middlesex. Dec. 27.

Assistant Road Foreman (£130), West Ham T.C. Mr. F. E. Hilleary, Town Clerk, West Ham. Dec. 27.

Chief Constable (£350), Lincoln T.C. Mr. W. T. Page, Town Clerk, Lincoln. Jan. 6.

Engineering Assistant (£110), Birkenhead T.C. Mr. C. Brownridge, Borough Engineer, Birkenhead. Jan. 4.

Health Visitor (£80), Wolverhampton Borough. Medical Officer of Health, Red Lion Street, Wolverhampton. Dec. 27.

Health Visitor (£80), Arnold U.D.C. Mr. C. F. Spencer, Clerk to the Council, Arnold, Notts. Dec. 28.

Inspector of Nuisances (£110), Thirsk R.D.C. Mr. W. Swarbreck, Clerk to the Council, Thirsk. Jan. 8.

Inspector of Nuisances (£120), Hemsworth R.D.C. Mr. J. Scholefield, Clerk to the Council, Hemsworth, near Wakefield. Jan. 2.

Nurse (£26), Llandaff and Dinas Powis Rural District Council Isolation Hospital. Mr. M. Warren, Clerk, Park House, Cardiff. Dec. 27.

Nurse (£30), Weston-super-Mare U.D. Council. S. C. Smith, Clerk, Town Hall, Weston-super-Mare. Jan. 2.

Nurse (£36), Epsom U.D. Council Isolation Hospital. E. G. Wilson, Clerk, Church Street, Epsom. Jan. 15.

Second Charge Nurse (£27), Leicester Borough Asylum. Medical Superintendent of the Asylum, Leicester.

Town Clerk (£500), Taunton T.C. Mr. G. H. Kite, Town Clerk, Taunton. Dec. 31.

### Miscellaneous Vacancies.

### Appointments Vacant.

Correspondence Clerk (£2 weekly), St. Helens Education Committee. The Secretary for Education, St. Helens.

Health Visitor and School Nurse (£104), Leicestershire County Council. W. J. Freer, Clerk, 10 New Street, Leicester. Jan. 2.

Housekeeper (£50), South Yorks Asylum. Medical Superintendent of the Asylum, Wadsley, near Sheffield. Dec. 29.

Junior Assistant Matrons (day and night) (£40) and Nurses, Middlesex County Asylum, Napsbury, St. Albans. Medical Superintendent.

Nurse (£70), York Education Committee. Mr. J. H. Mason, Secretary, Education Offices, Clifford Street, York. Dec. 27.

Railway Supervisor (£400), Port of London Authority. The Secretary, 109 Leadenhall Street, E.C. Dec. 30.

School Nurse (£70), City of York Education Committee. Mr. J. H. Mason, Secretary, Education Offices, Clifford Street, York. Dec. 27.

School Nurse and Health Visitor (£85), Borough of Bromley. Mr. F. Thackeray, Town Clerk, Morley, Yorks. Dec. 28.

Secretary (£175), Grantham Education Committee. Mr. A. H. Malim, Clerk to the Committee, Grantham.

Veterinary Inspector, Wilts C.C. Mr. R. W. Merriman, Clerk to the Council, Trowbridge. Dec. 31.

### Situations Vacant

Cook (£34), Stafford County Asylum. Medical Superintendent of the Asylum, Stafford.

